# Concrete Crack Detection in Extremely Dark Environments Based on Infrared-Visible Multi-Level Registration Fusion and Frequency Decoupling

**DOI:** 10.3390/s26092612

**Published:** 2026-04-23

**Authors:** Zixiang Li, Weishuai Xie, Bingquan Xiang

**Affiliations:** 1 School of Civil Engineering and Architecture, Anhui University of Science and Technology, No. 168 Tai Feng Road, Tianjia’an District, Huainan 232001, China; 2024200538@aust.edu.cn; 2 Anhui Provincial Key Laboratory of Building Earthquake Disaster Mitigation and Green Operations, Anhui Institute of Building Research & Design, Hefei 230001, China; xbqjiankeyuan@163.com

**Keywords:** extremely dark environments, heterogeneous registration, multi-modal fusion, crack segmentation, frequency decoupling

## Abstract

To address the issues of difficult heterogeneous image registration and low segmentation accuracy caused by the severe lack of illumination and significant modal differences in concrete cracks in extremely dark environments, this paper proposes a two-stage processing framework of registration–fusion first, and decoupling–segmentation later. In the registration and fusion stage, a registration algorithm based on morphological priors and multi-level quadtree spatial constraints is designed. This approach transforms the problem from pixel grayscale matching to spatial topological matching, achieving a feature fusion of high infrared saliency and high visible light sharpness. In the segmentation stage, a Latent Frequency-Decoupled Topological Network (LFDT-Net) is proposed. It utilizes Discrete Wavelet Transform (DWT) to achieve high-fidelity frequency decoupling of the low-frequency infrared backbone and the high-frequency visible light edges. Furthermore, a Cross-Frequency Guidance Module is utilized to eliminate double-edged artifacts, and a skeleton-aware topological loss function is introduced to constrain the topological integrity of the cracks. Experimental results on a self-built heterogeneous multi-modal crack dataset demonstrate that the proposed method significantly outperforms existing mainstream methods in registration accuracy, fusion quality, and segmentation accuracy. Achieving a mean Intersection over Union (mIoU) of 81.7%, the method effectively suppresses background noise in dark environments and precisely restores the microscopic edges and continuous topological structures of faint cracks.

## 1. Introduction

As the core carriers of modern infrastructure, the precise detection and quantitative analysis of surface cracks on concrete structures are critical technical aspects for ensuring structural safety, evaluating service performance, and implementing preventive maintenance [1,2,3]. In typical service scenarios such as roads [4], bridges [5], tunnels [6], and underground pipe networks [7], limited by lighting conditions, numerous inspection tasks must be carried out at night with low foot traffic or in environments with severe illumination deficiency. However, extremely dark environments pose severe challenges to apparent machine vision-based structural defect detection: the signal-to-noise ratio of conventional visible images drops sharply, and crack textures are submerged by background noise, making them difficult to use directly for effective identification [8].

Infrared imaging technology offers a new possibility to resolve these dilemmas. As a non-contact, passive sensing method, infrared thermography forms images by capturing thermal radiation differences on the object’s surface, demonstrating a natural immunity to lighting conditions [9]. In dark environments, because the internal voids of concrete cracks are filled with air, their thermal conductivity differs significantly from that of intact concrete. This causes the crack regions in thermal imaging to exhibit unique temperature field distribution characteristics, thereby outlining the approximate contours of the cracks. This characteristic has made infrared imaging one of the most widely applied mainstream technologies in nocturnal structural inspections [10].

There have been many explorations, as well as certain progressions made in crack detection using infrared imaging technology in extremely dark environments. Kazuma Shibano et al. utilized a random forest algorithm to select useful explanatory features to confirm the impact of infrared images on crack detection [11]. Jiang, J. et al. tested their proposed lightweight network GSkYOLOv5 using a self-built infrared dataset of asphalt pavement cracks, proving its superiority over other algorithms [12]. Gu, W. et al. employed a control variable method to evaluate the feasibility and applicable conditions of using infrared thermography to detect lining crack defects [9]. Although infrared thermography can outline thermal field contours of cracks based on thermal radiation differences, it commonly suffers from blurred edges, insufficient contrast, and nonlinear geometric distortions due to thermal diffusion effects and physical lens limitations [13]. To address this, many researchers have proposed fusing infrared and visible images to obtain fused representations for subsequent processing. For example, Yingnan Geng et al. proposed a scene information embedding (SIE) algorithm to represent the sufficiency of image features for scene information, which was then embedded into a CNN baseline model to solve the loss of background information in deep features extracted by neural networks [14]. Feiyan Cheng et al. designed a modality translation network equipped with a dual-branch multi-scale feature module (DBMS) and a feature selection module (FSM) to enhance feature representation and facilitate the selection of discriminative features for accurate image alignment [15].

Recently, to address the severe challenge of computer vision tasks in low-light environments, numerous state-of-the-art (SOTA) enhancement and detection methods have emerged, which can be broadly categorized into single-modal enhancement and dual-modal fusion paradigms. In the single-modal domain, recent advanced architectures aim to restore visibility by amplifying weak illumination features. For instance, Pu et al. [16] proposed FSRNet, employing a Frequency-Space Recovery Network to amplify high-frequency defect components on indoor concrete walls. Similarly, Yan et al. [17] designed PromptHDR, utilizing Transformer-based prompt learning and Mamba mechanisms to mitigate color distortion in underground mines. While highly effective in dimly lit scenes, the core distinction between these single-modal SOTA approaches and our present study lies in the fundamental physical limits of RGB data. In the extremely dark environments discussed in this paper—where the signal-to-noise ratio approaches zero—single-modal algorithms inevitably amplify massive random environmental noise alongside faint targets, failing to reconstruct the physically lost crack topology.

To bypass single-modal limitations, recent studies have actively explored dual-modal (infrared and visible) fusion for crack detection. For example, Liu et al. [18] utilized infrared-visible fusion combined with CNNs to accurately classify the severity levels of fatigue cracks. Moving towards automated localization, Chen et al. [19] proposed YOLOv11-DCFNet with a Cross-Modality Fusion Transformer for macroscopic bounding-box detection of road cracks under no-light conditions. For pixel-level tasks, Shi et al. [20] introduced a dynamic sparse attention mechanism applied to guided-filtered fused images to segment nighttime pavement cracks. However, a critical architectural distinction exists between these existing dual-modal approaches and our study. Most current dual-modal networks focus on image-level classification or macroscopic object detection, lacking the capacity for fine-grained pixel-level topological extraction. Even in recent pixel-level segmentation models [20], the fused multi-modal data are processed within a coupled spatial domain. In extremely dark scenarios, this coupled spatial processing inevitably causes severe thermal diffusion (originating from the infrared modality) to swallow and blur faint, microscopic physical edges (originating from the visible modality). Consequently, isolating this cross-modal interference requires a fundamental paradigm shift from coupled spatial processing to physics-driven frequency decoupling. Mathematically separating the macroscopic thermal background (low frequency) from sharp physical edges (high frequency) at the source has become the key to perfectly preserving the microscopic topological continuity of fine cracks in extreme darkness.

However, in the aforementioned studies, it is presumed that visible images have sufficient brightness and contrast to extract stable and repeatable local features (e.g., edges, corners) for registration with infrared images. In extremely dark environments, this premise is difficult to hold true. In such conditions, the signal-to-noise ratio of visible images is exceedingly low, and texture information is almost obliterated by noise. Traditional feature point matching algorithms (such as SIFT [21] and RIFT [22]) and registration networks relying on deep feature extraction both fail due to the inability to obtain reliable feature correspondences. This leads to the failure of spatial alignment between infrared and visible images at the pixel level, making the subsequent fusion process impossible. Therefore, achieving precise registration of heterogeneous images in extremely dark environments without relying on local gradient features is a major technical obstacle for the effective alignment of cross-modal data in practical multi-sensor visual inspections.

To address the aforementioned challenges, this paper proposes a two-stage processing framework of registration–fusion first, and decoupling–segmentation later. First, at the pre-processing level, we discard the reliance on local gradients and propose a heterogeneous image registration and fusion algorithm based on morphological priors and multi-level quadtree spatial constraints. This algorithm strips common crack structures through morphological transformations, shifting the registration problem into spatial topological matching. Combined with a multi-level quadtree, it achieves adaptive deformation correction from macro to micro scales, generating a comprehensive feature map that possesses both infrared saliency and visible light sharpness. Secondly, in the segmentation stage, targeting the frequency heterogeneity of the fused image, a Latent Frequency-Decoupled Topological Network (LFDT-Net) is designed. This network utilizes Discrete Wavelet Transform to achieve high-fidelity frequency decoupling of the low-frequency backbone and high-frequency edges. It employs a Cross-Frequency Guidance Module to eliminate double-edged artifacts and introduces a skeleton-aware topological loss function to maintain the mechanical continuity of the cracks. Experimental results demonstrate that both the registration accuracy and segmentation performance of this method on the self-built extremely dark environment dataset are significantly superior to existing mainstream methods.

## 2. Heterogeneous Image Registration and Fusion Based on Morphological Priors and Multi-Level Quadtree Spatial Constraints

To solve the problem of registering and fusing infrared and visible images in dark environments, a novel heterogeneous image registration and fusion architecture is proposed. This method abandons the reliance on local pixel gradients and instead utilizes morphological spatial prior structures for feature extraction. Furthermore, it introduces a multi-level quadtree spatial constraint mechanism to achieve adaptive deformation registration and ultimately accomplishes high-quality image fusion through cross-modal high-frequency detail injection.

### 2.1. Feature Extraction Based on Morphological Spatial Structures

In extremely dark environments, cracks in visible images typically appear as faint, continuous, and elongated dark structures that are surrounded by random environmental noise. In contrast, cracks in infrared images present as bright thermal signal bands with higher global contrast. To bridge the modal differences between the two, this paper first employs mathematical morphology to independently extract the structural features of both modalities in the spatial domain.

Let the input original image be I(x,y), and the structuring element be B(u,v). In this paper, to accommodate the random and omnidirectional growth characteristics of concrete cracks, B(u,v) is defined as an isotropic disk-shaped (circular) matrix. Furthermore, considering that the physical width of micro-cracks at a 500 × 500 resolution typically ranges from 2 to 5 pixels, the radius of the disk is empirically set to 3 pixels (forming a 7 × 7 local receptive field). This specific size ensures that the structuring element is slightly wider than the crack target, effectively isolating the fine crack topology from large-scale background illumination variations. Sensitivity analysis reveals that the morphological extraction quality is highly dependent on this scale matching. Using a smaller radius (e.g., radius ≤2) causes the structuring element to fall entirely within wider crack segments, leading to incomplete extraction and severe topological fragmentation. Conversely, a significantly larger radius (e.g., radius ≥5) inadvertently captures large-scale uneven background illumination and thermal diffusion artifacts as false-positive foreground noise. Thus, a radius of 3 optimally balances crack integrity and noise suppression.

For the extremely dark visible image $I_{vis}$, this paper employs the morphological black-hat transform [23] specifically to extract subtle groove structures that are darker than the structuring element, while simultaneously suppressing the overall dark background. Its mathematical expression is as follows:

(1) $$T_{black}(I_{vis})=(I_{vis}•B)-I_{vis},$$

where • denotes the morphological closing operation. By filling the cracks through the closing operation and then subtracting the original image, the initially faint black cracks can be transformed into a prominent, bright, high-frequency feature map.

For the infrared thermography image $I_{ir}$, to extract the highly luminous main contours of the cracks and suppress background thermal noise, this paper applies the morphological top-hat transform [24]:

(2) $$T_{top}(I_{ir})=I_{ir}-(I_{ir}\circ B),$$

where ∘ denotes the morphological opening operation.

After the aforementioned morphological prior extraction, the massive differences in absolute pixel grayscale between the heterogeneous images are eliminated. Both are mapped into binarized spatial distribution sets that are dominated by crack topological structures, laying the foundation for subsequent spatial registration.

### 2.2. Multi-Level Quadtree Spatial Topological Constraints and Adaptive Registration

After extracting the spatial topological structures of the heterogeneous images, a robust registration algorithm is required to align the multi-modal features. While data-driven methods like Optical Flow and deep feature-matching networks (e.g., SuperGlue) excel in standard tasks, they fail fundamentally in extremely dark cross-modal scenarios. Optical flow violates the cross-modal brightness constancy assumption, and deep registration networks struggle with zero-texture, intensity-inverted image pairs (e.g., bright IR signals versus dark visible grooves), frequently causing catastrophic matching failures on noise. Therefore, to bypass unreliable pixel-level intensities and textures, this paper proposes a physically driven multi-level quadtree spatial constraint registration algorithm. By directly extracting the macroscopic topological skeletons, this algorithm simulates a cell-division-like approximation process from a global macroscopic scale to a local microscopic scale, effectively avoiding local optimal solutions.

#### 2.2.1. Spatial Subdivision and Local Centroid Extraction

Let the extracted binarized feature maps of visible light and infrared be $F_{vis}$ and $F_{ir}$, respectively. The algorithm starts from level 0 (the global image) and divides the image space with a $2^{d}\times 2^{d}$ grid, where d represents the current quadtree depth.

Within any sub-quadrant region $R_{k}$, the zero-order moment and first-order spatial moments of this region are calculated:

(3) $$m_{pq}=\iint_{R_{k}}x^{p}y^{q}F(x,y)dxdy,$$

If and only if the zero-order moments of both $F_{vis}$ and $F_{ir}$ within the same sub-quadrant $R_{k}$ are greater than zero (i.e., $m_{00}^{vis}>0$ and $m_{00}^{ir}>0$, indicating that both modalities possess crack topology within this local spatial region), their geometric centroids within this sub-region are calculated as the control points for the current level:

(4) $$x_{c}=\frac{m_{10}}{m_{00}},\;y_{c}=\frac{m_{01}}{m_{00}},$$

#### 2.2.2. Downsampling Mechanism and Global–Local Constraints

To enhance the robustness of the registration, the algorithm introduces a downsampling mechanism at shallow layers (e.g., d=1,2). At this stage, the image is compressed to an extremely low resolution (e.g., 2×2 or 4×4 pixel blocks), which effectively shields the high-frequency interference caused by infrared edge burrs and visible light noise, establishing rough topological connections of general light and dark quadrants only at the maximum scale. As the depth d gradually increases (up to d=8), the spatial grid is subdivided exponentially. It is worth noting that the maximum depth d=8 is mathematically derived from the 500×500 input resolution to balance registration accuracy and computational efficiency. At d=8, the space is partitioned into $2^{8}\times 2^{8}=256\times 256$ grids, meaning each sub-quadrant represents a physical area of approximately 2×2 pixels. This is the optimal minimal neighborhood required to calculate a valid local spatial centroid (avoiding single-pixel noise), ensuring sub-pixel registration accuracy for micro-cracks. Conversely, if d≥9 ($2^{9}=512$), the grid resolution would exceed the image’s physical resolution limit, creating massive empty quadrants ($m_{00}=0$). This would not only trigger division-by-zero errors in centroid computations but also exponentially increase the computational burden with zero precision gain. Thus, bounded by d=8, the centroid control points grow densely like neural synapses, ultimately locking the mapping relationship between the deformed infrared cracks and the true visible cracks seamlessly at the pixel level.

By collecting the set of high-confidence centroid pairs $P={\left\{(p_{vis}^{\left(i\right)},p_{ir}^{\left(i\right)})\right\}}_{i=1}^{N}$ at depths d≥3, the algorithm employs the RANSAC method [25] to robustly estimate the homography matrix H, forcibly aligning the infrared image $I_{ir}$ into the spatial coordinate system of the visible image. Given that the camera’s macroscopic working distance (typically 1–2 m) far exceeds the millimeter-scale depth variations in the micro-cracks, the localized concrete surface is mathematically approximated as a piecewise 2D plane. This rigorously justifies the planar transformation assumption inherent in the homography matrix estimation:

(5) $$\left[\begin{array}{c} x_{vis}\\ y_{vis}\\ 1 \end{array}\right]=H\left[\begin{array}{c} x_{ir}\\ y_{ir}\\ 1 \end{array}\right],$$

### 2.3. Cross-Modal Image Fusion

After the deformation correction is completed, the infrared image provides extremely stable target saliency, while the visible image contains accurate high-frequency textures. This paper adopts a weighted fusion method based on structural high-frequency extraction.

Let the deformation-aligned infrared grayscale image be $G_{ir\_warped}$, and the high-frequency detail map of the visible grayscale image be $E_{vis}$. The final fused image $I_{fused}$ is calculated by the following formula:

(6) $$I_{fused}(x,y)=\alpha \cdot G_{ir\_warped}(x,y)+\beta \cdot E_{vis}(x,y),$$

where α and β are the fusion weight coefficients. The determination of these coefficients is crucial for balancing infrared saliency and visible light sharpness. If α is too large, fusion will suffer from thermal overexposure; if β is insufficient, the faint visible edges will fail to sharpen the blurred thermal boundaries. To scientifically determine the optimal parameter combination, an ablation study was conducted on the dataset using three objective Image Quality Assessment (IQA) metrics: Average Gradient (AG), Spatial Frequency (SF), and Information Entropy (EN). AG and SF reflect the clarity and microscopic detail retention of the image, while EN evaluates the richness of the overall structural information. The quantitative results are presented in Table 1.

As shown in Table 1, the parameter pair (α=0.6,β=2.5) achieves optimal performance across all quantitative metrics. Physically, α=0.6 effectively compresses the global thermal diffusion noise of the infrared base map, while the amplification factor β=2.5 perfectly endows the faint visible high-frequency details with enough intensity to physically slice and sharpen the blurred thermal edges without inciting severe salt-and-pepper background noise. Therefore, these values were fixed as the optimal configuration for generating the comprehensive feature maps for the downstream LFDT-Net.

**Figure 1:**
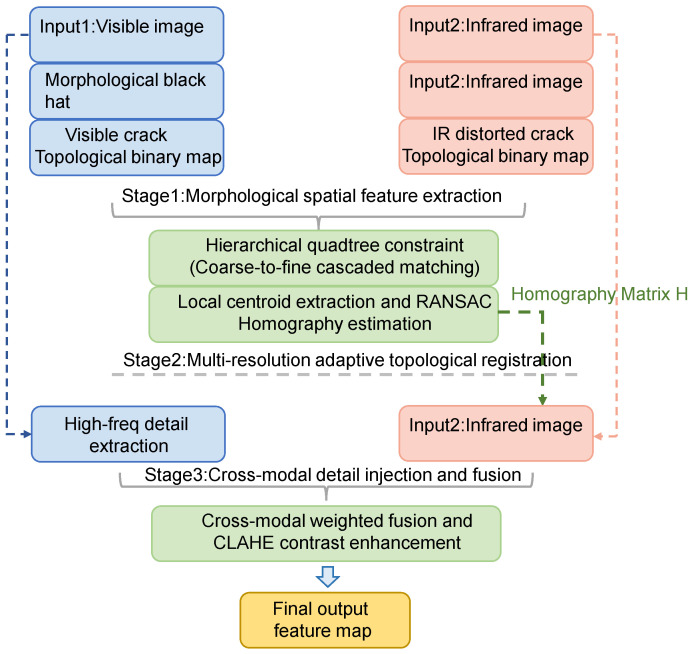
Flowchart of the infrared and visible image registration and fusion algorithm.

Finally, Contrast Limited Adaptive Histogram Equalization (CLAHE) is utilized to further enhance the visual contrast, outputting a high-quality, comprehensive feature map that can be directly used for high-precision downstream segmentation tasks. The entire process of the algorithm is shown in Figure 1.

### 2.4. Dynamic Evolution Visualization of Multi-Resolution Cascaded Matching

**Figure 2:**
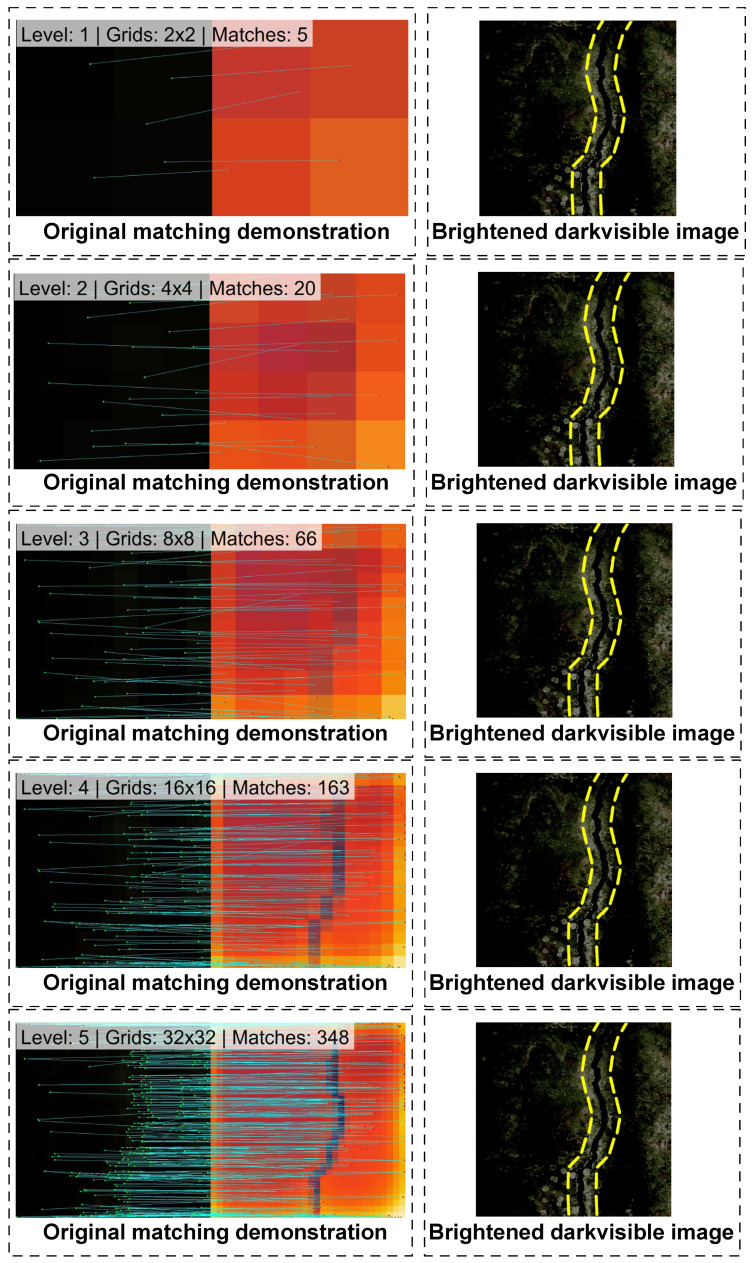

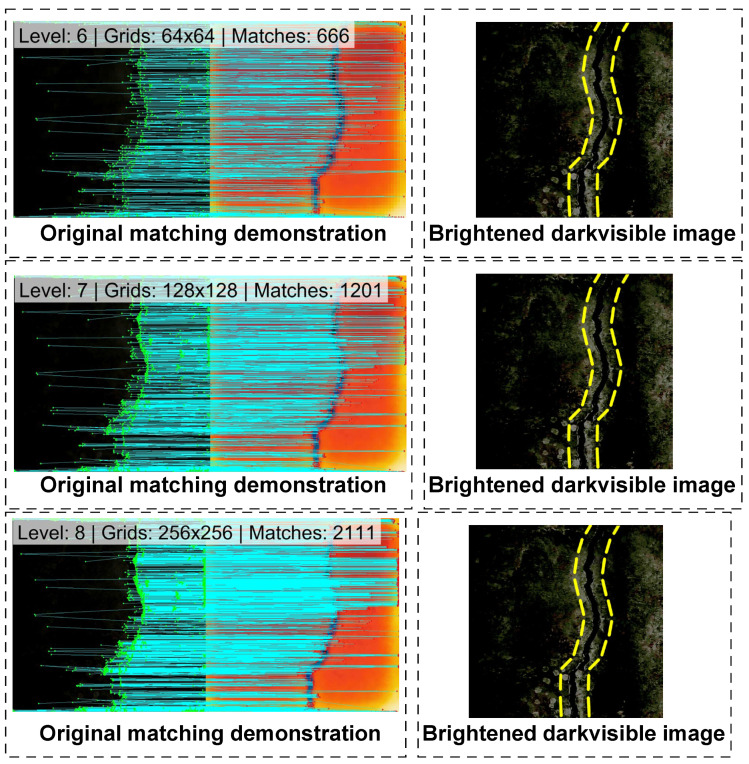
Dynamic evolution process of multi-resolution cascaded matching.

Through the dynamic evolution of the multi-resolution cascaded matching demonstrated in Figure 2, the effectiveness of the proposed multi-level quadtree spatial topological constraint mechanism in overcoming extreme deformation and massive noise can be intuitively verified. To explicitly address the visual discernibility challenge caused by the dense connectivity lines at higher levels, a comprehensive dual-frame comparative layout is implemented across all eight evolutionary stages. For each level, the first frame presents the original matching demonstration, showing the algorithm operating directly on the raw, extremely dark visible image and the corresponding infrared image, and the adjacent frame presents a brightened image with ground truth outline (yellow dashed lines) verification. Here, Gamma correction is applied to the extremely dark visible image to clearly reveal the tortuous concrete crack structure, and this intricate skeleton is explicitly outlined with an eye-catching yellow dashed line.

In shallow layers (such as Depth 1 and Depth 2), the images are forcibly downsampled into macroscopic mosaic grids [e.g., of 2×2 and 4×4]. At this extremely low resolution, the complex environmental noise in the visible image and the thermal diffusion burrs in the infrared image are both obliterated by spatial averaging in a physical sense. At this moment, the algorithm only captures the global distribution of light and dark quadrants, establishing the initial macroscopic topological connections.

As the evolution depth increases (from Depth 3 to Depth 5), the spatial grid splits exponentially, and the local textures of the images begin to emerge. At this stage, the macroscopic topological constraints from the previous level forcibly eliminate the possibility of erroneous matching. The centroid control lines begin to densely proliferate along the actual crack orientation, and the algorithm smoothly transitions from finding quadrants to locking onto contours.

When the depth advances to Depth 8, the mosaic effect completely fades away, and the images are restored to their original true high-definition resolution. At this point, the control point pairs generated by the multi-level quadtree are densely and precisely distributed along the edges of the entire crack skeleton. This coarse-to-fine evolution process intuitively proves that by strongly coupling the entropy increase in image resolution with the spatial approximation scale, the proposed algorithm successfully bridges the gap in the underlying pixel distribution of heterogeneous images, achieving a nearly seamless nonlinear deformation binding.

**Figure 3:**
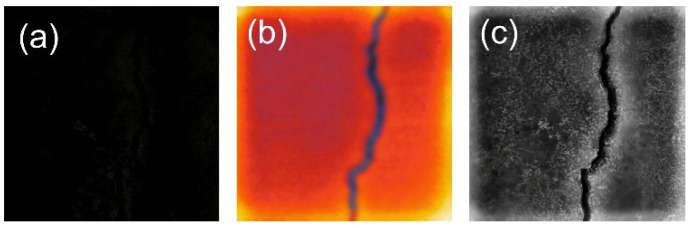
Fusion results: (**a**) original visible image in a dark environment; (**b**) infrared image; (**c**) fused result.

The photographs taken in the extremely dark environment, the corresponding infrared images, and the fused images are shown in Figure 3a, Figure 3b, Figure 3c, respectively. It can be seen that in the original visible image (Figure 3a), the crack structure is almost completely submerged by environmental noise; the signal-to-noise ratio is extremely low, making it difficult for the human eye to effectively identify the orientation and boundaries of the crack. While the original infrared image (Figure 3b) can outline the approximate region of the crack relying on thermal radiation differences, the edges of the crack exhibit obvious blurring and burrs due to the thermal diffusion effect, leading to geometric distortion.

The image processed by the fusion algorithm proposed in this paper (Figure 3c) achieves the following complementary advantages: the global thermal radiation distribution provided by the infrared image is completely preserved, endowing the crack region with a stable, high-saliency background; meanwhile, the high-frequency details extracted and injected from the visible image perform physical-level sharpening on the blurred infrared edges, making the main crack clear and coherent, and precisely restoring subtle textures.

To rigorously validate the visual improvements and address the necessity of objective performance metrics, we further conducted a comprehensive quantitative evaluation on both the registration accuracy and fusion quality across the dataset, as summarized in Table 2.

As indicated in Table 2, the quadtree spatial constraint registration achieves an excellent average RMSE of 2.24 pixels. This objectively demonstrates that the algorithm successfully overcomes the severe spatial parallax and modal gaps, locking the cross-modal centroids with near-pixel-level precision.

In terms of fusion quality, the fused images yield a high Information Entropy (EN) of 6.67, proving that the multi-modal integration strategy successfully injects rich structural details into the final output. Furthermore, the structural fidelity exhibits highly logical asymmetric SSIM scores. The high SSIM (0.92), relative to the IR modality, confirms that the macroscopic thermal morphology is perfectly preserved as the base framework. Conversely, the seemingly lower SSIM (0.45), relative to the visible modality, is physically expected and highly desirable: the original visible images are severely degraded by extensive dark background noise. The fusion algorithm strictly extracts only the sparse, high-frequency microscopic crack edges while aggressively discarding the vast, useless dark background. Thus, this asymmetric structural similarity objectively validates our feature-stripping fusion design, providing a high-quality data foundation for subsequent automated identification.

## 3. Latent Frequency-Decoupled Topological Segmentation Network (LFDT-Net) for Multi-Modal Heterogeneous Chimeric Features

Through the morphological spatial constraint registration and fusion in Section 2, a comprehensive feature map $I_{fused}\in {\mathbb{R}}^{H\times W\times 1}$ with high saliency and high sharpness is obtained. However, this feature map exhibits strong separated frequency heterogeneity in its physical mechanism: its low-frequency energy (macroscopic orientation and global contrast) originates from infrared thermal radiation, while its high-frequency details (pixel-level sharp edges) are injected by visible light. In traditional Convolutional Neural Networks (CNNs) such as U-Net or DeepLabV3+, the downsampling modules (typically max pooling or Strided Convolution) act as low-pass filters, which obliterate the high-frequency visible light edges in the fused image [26]. Furthermore, heterogeneous disparity at the microscopic scale easily triggers double-edge artifacts, causing conventional cross-entropy loss functions to produce gradient oscillations at the boundaries, which render the predicted cracks jagged or topologically fractured.

To address the aforementioned challenges, this paper abandons general semantic segmentation methods and proposes a tailor-made Latent Frequency-Decoupled Topological Network (LFDT-Net). This network takes frequency dual-stream decoupling as its backbone, utilizes cross-guidance to eliminate artifacts, and introduces a skeleton-aware topological loss function based on the continuity of mechanical stress release, achieving precise decoding of heterogeneous chimeric features.

### 3.1. Overall Architecture Design of LFDT-Net

**Figure 4:**
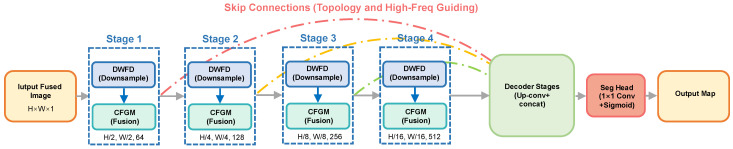
Architecture of the LFDT-Net.

LFDT-Net adopts an encoder–decoder architecture, as shown in Figure 4, but completely reconstructs the feature extraction and transmission mechanisms. The initial resolution of the input image $I_{fused}$ is H×W×1. The encoder contains four levels of decoupled downsampling stages (Stages 1–4). Instead of utilizing conventional pooling, each level rigorously splits the features into a low-frequency stream $X_{low}$ and a high-frequency stream $X_{high}$ through a Discrete Wavelet Frequency Decoupling (DWFD) module.

As the network deepens, the spatial dimensions of the feature maps sequentially decrease by a factor of 1/2. The resolution changes are as follows:

Stage 1: $\frac{H}{2}\times \frac{W}{2}\times C_{1}$

Stage 2: $\frac{H}{4}\times \frac{W}{4}\times C_{2}$ ($C_{2}=128$)

Stage 3: $\frac{H}{8}\times \frac{W}{8}\times C_{3}$ ($C_{3}=256$)

Stage 4: $\frac{H}{16}\times \frac{W}{16}\times C_{4}$ ($C_{4}=512$)

Within each stage, the dual-stream features are fed into the Cross-Frequency Guidance Module (CFGM) for information interaction and artifact elimination. Subsequently, channel fusion is performed before passing the features to the next layer. In the decoder stage, upsampling is performed via bilinear interpolation, followed by skip connections with the fused features from the corresponding levels of the encoder. Finally, a 1×1 convolution is applied to output a binarized crack prediction probability map with dimensions of H×W×1.

To explicitly demonstrate the layer-by-layer feature transformations, tensor dimensions, and operator configurations of the proposed network, the detailed architectural parameters of LFDT-Net are summarized in Table 3. Assuming the input fused image size is H×W×1, the network incrementally expands the channel capacity while physically decoupling and refining the features at each downsampling stage.

### 3.2. Discrete Wavelet Frequency Decoupling Downsampling Module (DWFD)

To effectively retain the high-frequency edges injected by visible light during the downsampling process, and to avoid the problem of traditional strided convolutions or max pooling obliterating image edge details, LFDT-Net introduces the 2D Discrete Wavelet Transform (2D-DWT, adopting the Haar wavelet basis) to replace max pooling. The selection of the Haar wavelet over other complex wavelet families (e.g., Daubechies) is primarily driven by its strict spatial compatibility with CNN downsampling operations. Specifically, the Haar wavelet possesses a minimal 2×2 spatial support footprint, which perfectly aligns with the conventional 2×2 window size of CNN pooling layers. This intrinsically avoids the spatial-phase shifting and boundary padding artifacts that longer wavelets (e.g., Db2 or Db4 with 4×4 and 8×8 supports) would introduce into deep feature maps. Furthermore, the Haar operators consist of simple normalized {1,−1} weights, requiring only basic addition and subtraction. This maximizes the hardware computational efficiency (maintaining high FPS) while effectively completing the frequency-domain decoupling.

Let the input feature map of the i-th layer be $X^{\left(i\right)}\in {\mathbb{R}}^{H_{i}\times W_{i}\times C_{i}}$. The 2D-DWT utilizes four different filters (low-pass $f_{L}$ and high-pass $f_{H}$) to perform convolution operations with a stride of 2 on the feature map, outputting four sub-band features with dimensions of $\frac{H_{i}}{2}\times \frac{W_{i}}{2}\times C_{i}$: LL (low frequency), LH (horizontal high frequency), HL (vertical high frequency), and HH (diagonal high frequency). The mathematical expressions are as follows:

(7) $$\begin{array}{c} LL=(X^{\left(i\right)}\times f_{LL})↓2,\;LH=(X^{\left(i\right)}\times f_{LH})↓2,\\ HL=(X^{\left(i\right)}\times f_{HL})↓2,\;HH=(X^{\left(i\right)}\times f_{HH})↓2, \end{array}$$

where $f_{LL}$, $f_{LH}$, $f_{HL}$, and $f_{HH}$ represent the low-pass filter, horizontal high-pass filter, vertical high-pass filter, and diagonal high-pass filter, respectively. ↓ denotes downsampling.

During the forward propagation of the network, these four operators perform grouped convolutions on the input tensor. Among them, $f_{LL}$ acts as an averaging operator, comprehensively extracting the macroscopic thermal radiation energy distribution of the infrared stream; meanwhile, the other three high-pass operators precisely strip the high-frequency edge contours of the visible light stream in different gradient directions by calculating the spatial differences in adjacent pixels.

The LL sub-band containing abundant infrared background energy is directly defined as the low-frequency stream ($X_{low}^{\left(i\right)}=LL$). Meanwhile, the three high-frequency sub-bands containing the physically sliced visible light edges are concatenated along the channel dimension and reduced in dimension via a 1×1 convolution to be defined as the high-frequency stream:

(8) $$X_{high}^{\left(i\right)}={\mathrm{Conv}}_{1\times 1}(Concat(LH,HL,HH)),$$

**Figure 5:**
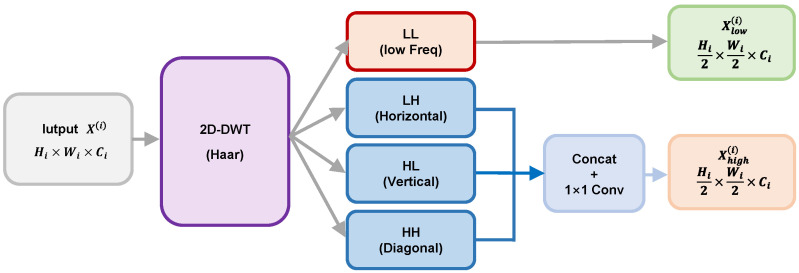
Schematic diagram of the DWFD module.

Through this mechanism, the feature map not only completes downsampling in the spatial scale but is also forcibly split into two paths with clear independent semantics—the infrared backbone and the visible light edges—in terms of physical mechanism, thereby eliminating the attrition of sharp edges caused by downsampling at the source. The schematic diagram of the DWFD module is shown in Figure 5.

### 3.3. Cross-Frequency Guidance Module (CFGM)

After obtaining the information-preserving downsampled dual-stream features, to solve the double-edge artifacts (Micro-Ghosting) caused by microscopic parallax, a Cross-Frequency Guidance Module (CFGM) is designed. Unlike conventional approaches that simply stack parallel spatial and channel attention, CFGM adopts an asymmetrical sequential cross-guidance mechanism. This specific two-stage sequential design is strictly dictated by the physical characteristics of the heterogeneous features in dark environments: denoising must strictly precede sharpening.

The high-frequency visible stream contains sharp physical edges but is heavily polluted by random environmental noise. If the network were to utilize the high-frequency stream to guide the low-frequency backbone directly, the unpurified visible noise would pollute the clean thermal topology, causing massive false-positive segmentations. Therefore, CFGM allows the high- and low-frequency features to mutually guide each other by applying strict physical constraints through a specifically ordered two-step strategy.

In the first stage of the module, the primary purpose is to filter background noise through a low frequency guiding high frequency. The high-frequency visible edges extracted in extremely dark environments are often accompanied by artifact noise. The low-frequency stream $X_{low}^{\left(i\right)}$ contains the main structure of the crack, which lacks details but is relatively pure. It is utilized to generate a spatial saliency mask $M_{low}$ to suppress the pseudo-noise activated by the high-frequency stream in the background regions:

(9) $$M_{low}=\sigma ({\mathrm{Conv}}_{3\times 3}(X_{low}^{\left(i\right)})),$$

where σ is the Sigmoid activation function.

Subsequently, $M_{low}$ is multiplied element-wise with the high-frequency stream to forcibly filter out the complex noise that is activated in the background regions, outputting the refined high-frequency stream $X_{high\_refined}^{\left(i\right)}$:

(10) $$X_{high\_refined}^{\left(i\right)}=X_{high}^{\left(i\right)}⊗M_{low},$$

where ⊗ denotes element-wise multiplication.

The main purpose of the second stage is to achieve the slicing of physical edges through a high frequency guiding a low frequency. The high-frequency stream $X_{high}^{\left(i\right)}$ contains sharp true physical boundaries. It is utilized to generate an edge-sharpening matrix $E_{high}$, which is superimposed onto the low-frequency stream as a residual gain to cut off the thermal diffusion-induced blurring at the edges of the low-frequency stream:

(11) $$E_{high}=\sigma ({\mathrm{Conv}}_{3\times 3}(X_{high\_refined}^{\left(i\right)})),$$

It is worth noting that if $E_{high}$ is directly multiplied into the low-frequency stream, it will lead to the loss of the original global thermal saliency base map of the infrared features in non-edge flat regions (i.e., where $E_{high}\approx 0$). Therefore, this paper introduces a residual amplification operator $1+E_{high}$. The calculation formula for the refined low-frequency stream is defined as:

(12) $$X_{low\_refined}^{\left(i\right)}=X_{low}^{\left(i\right)}⊗(1+E_{high}),$$

The physical significance of this formula lies in the flat background regions where $E_{high}\approx 0$ and the multiplier is 1, perfectly preserving the low-frequency infrared background. In contrast, at the extremely narrow crack edges ($E_{high}\approx 1$), the multiplier approaches 2. This is equivalent to feeding back the high-frequency energy of visible light with doubled weight to slice off the thermal diffusion-blurred parts at the edges of the low-frequency stream.

After the cross-guidance in the previous two steps, the pure visible high-frequency stream and the sharp infrared low-frequency stream achieve high alignment in the spatial dimension. Finally, the refined dual-stream features are concatenated and fused for output to the next network level:

(13) $$X^{\left(i+1\right)}={\mathrm{Conv}}_{3\times 3}(Concat(X_{low\_refined}^{\left(i\right)},X_{high\_refined}^{\left(i\right)})),$$

**Figure 6:**
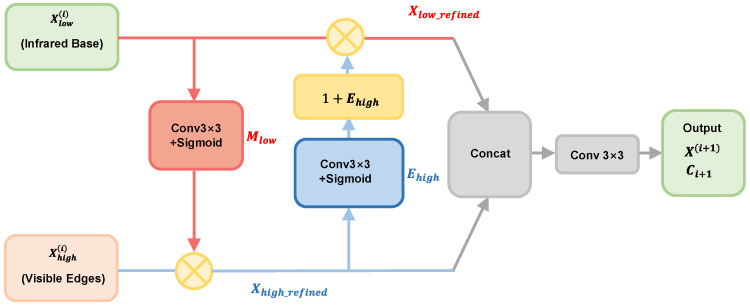
Schematic diagram of the CFGM.

The schematic diagram of the CFGM is shown in Figure 6.

### 3.4. Skeleton-Aware Topological Loss Function

Cracks generated in extremely dark and complex stress environments are, in their physical essence, continuous stress release channels. Conventional Binary Cross-Entropy (BCE) loss or Dice loss focuses only on isolated pixel-level classification accuracy and is extremely insensitive to topological fractures in the middle of a crack, even if they are only 1 pixel wide.

To endow the network with physical perception capabilities at the mechanical and geometric levels, this paper adaptively incorporates a skeleton-aware topological loss $L_{topo}$ (improved based on the clDice concept [27]) on top of conventional losses.

While the foundational mathematical concept of skeleton-based topological measurement originates from existing literature, this paper implements a specific engineering modification tailored for extremely dark environments. The theoretical motivation for this adaptation is driven by the fact that in dark environments with severe noise interference, early-epoch predictions of micro-cracks are exceptionally fragmented. This extreme fragmentation frequently leads to empty extracted skeletons, causing zero-denominator anomalies in standard topological calculations. Therefore, we formulated a numerically stabilized topological constraint:

Let the final predicted probability map of the network be $P\in {\left[0,1\right]}^{H\times W}$, and the ground truth label be $G\in {\left\{0,1\right\}}^{H\times W}$. First, a morphological soft-skeletonization algorithm [28] is utilized to extract the skeleton $S_{P}$ of the predicted map and the skeleton $S_{G}$ of the ground truth label, respectively. Subsequently, the Topological Precision $T_{prec}$ and Topological Sensitivity $T_{sens}$ of the skeletons are calculated:

(14) $$T_{prec}=\frac{\sum \left(S_{P}⊗G\right)}{\sum S_{P}},\;T_{sens}=\frac{\sum \left(S_{G}⊗P\right)}{\sum S_{G}},$$

The topological loss is defined as the reverse penalty of the harmonic mean of the two:

(15) $$L_{topo}=1-\frac{2\times T_{prec}\times T_{sens}}{T_{prec}+T_{sens}+\epsilon },$$

In Equation (15), the specific modification lies in the introduction of the micro-smoothing factor ϵ. This stabilization parameter guarantees that the gradients remain bounded and computable even when the network outputs completely disconnected noise in early training stages.

Finally, the total training loss function $L_{total}$ of LFDT-Net is jointly composed of three parts:

(16) $$L_{total}=L_{BCE}(P,G)+\lambda_{1}L_{Dice}(P,G)+\lambda_{2}L_{topo}(S_{P},S_{G},P,G),$$

where $\lambda_{1}$ and $\lambda_{2}$ are balance coefficients. This joint loss function forces the network not only to improve accuracy at the pixel level but also to ensure that the topology of the stress channels remains unfractured.

## 4. Construction and Pre-Processing of the Heterogeneous Multi-Modal Crack Dataset

### 4.1. Dataset Collection and Annotation

The image collection for this dataset primarily focused on typical extremely dark environments lacking illumination at night, such as highway pavements, buildings, and enclosed construction tunnels. The acquisition equipment included conventional RGB cameras and infrared thermal imagers. To ensure high data quality and the targeted nature of the algorithmic research, various complex crack morphologies were encompassed during the acquisition process. Following screening and elimination, a heterogeneous image database comprising 1000 sets of high-quality paired samples was ultimately constructed. Each sample set contained a visible image captured in an extremely dark environment and its corresponding infrared thermography image. The images were uniformly cropped and resized to a fixed resolution of 500×500. The annotation process was conducted using the LabelMe v4.5.13 software. To minimize subjective bias and ensure annotation consistency across the dataset, a rigorous multi-annotator cross-validation protocol was implemented. During the annotation process, by simultaneously referencing the registered high-contrast infrared base maps and the high-frequency visible detail maps, fine pixel-wise polygonal outlining was performed along the true physical edges of the cracks. Specifically, each image was independently annotated by two trained researchers. If the Intersection over Union (IoU) between their respective annotations fell below 90%, or if discrepancies arose regarding the topological connectivity of microscopic crack branches, a third senior structural engineering expert intervened to perform the final arbitration. This consensus mechanism effectively eliminated individual labeling deviations. Ultimately, the JSON files generated by LabelMe were batch-converted into 8-bit single-channel binarized mask images. In accordance with standard practices in the deep learning field, these 1000 sample sets were randomly and mutually exclusively divided at a ratio of 7:2:1. Specifically, the training set comprised 700 samples, the validation set contained 200 samples, and the testing set consisted of 100 samples. Although 1000 sample pairs may appear relatively limited compared to traditional single-modal open-source datasets (such as Crack500 or DeepCrack), LFDT-Net utilizes DWT as a mathematically deterministic prior so as to achieve lossless decoupling of high and low frequencies. This architecture fundamentally constrains the parameter search space and effectively reduces the network’s dependency on massive data scales.

### 4.2. Data Augmentation Strategy

**Figure 7:**
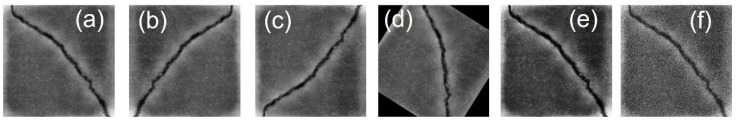
Examples of dataset augmentation: (**a**) original image; (**b**) horizontal flip; (**c**) vertical flip; (**d**) random rotation; (**e**) contrast alteration; (**f**) noise addition.

To enhance the network’s translation invariance and robustness against spatial deformations, illumination fluctuations, and sensor noise, various data augmentation strategies were applied to the training set during the training phase. Flipping operations were employed to simulate crack morphologies captured from different driving directions and viewing angles, eliminating the model’s memory dependence on specific crack growth directions. Random contrast adjustments were adopted to prevent the model from relying on absolute grayscale thresholds, compelling it to learn the essential morphological features of the cracks instead. Minor brightness variations were introduced to simulate slight light source fluctuations encountered under complex inspection conditions. Furthermore, noise was randomly superimposed onto the images to improve the model’s feature decoupling and anti-noise segmentation capabilities when confronting background interference, as shown in Figure 7.

## 5. Crack Recognition Experiments and Result Analysis in Dark Environments

### 5.1. Experimental Environment Configuration and Hyperparameter Settings

The experiments were conducted on a 64-bit Windows-based workstation. The Central Processing Unit (CPU) employed was a 12th Generation Intel^®^ Core™ i9-12900KF (Intel, Santa Clara, CA, USA), featuring a base frequency of 3.20 GHz and a 16-core architecture. A deep learning computational card with 24 GB of VRAM was selected as the Graphics Processing Unit (GPU). In terms of the software parallel computing architecture, CUDA 11.6 and the deep neural network acceleration library cuDNN 8.6 were deployed to provide hardware-level acceleration for underlying matrix multiplication and convolution operations. The core algorithm codes were entirely written based on the mainstream deep learning framework PyTorch v2.1.0. The specific global hyperparameter configurations are presented in Table 4.

### 5.2. Model Evaluation Metrics

To objectively and quantitatively evaluate the comprehensive performance of LFDT-Net and the comparison models in heterogeneous crack detection in dark environments from multiple dimensions, a comprehensive evaluation metric system encompassing segmentation accuracy and deployment efficiency was selected.

In terms of model accuracy evaluation, the mean Intersection over Union (mIoU), mean Pixel Accuracy (mPA), and F1-Score were adopted as evaluation metrics. As these are standard evaluation metrics widely utilized in semantic segmentation tasks, their canonical mathematical formulations are omitted here for conciseness. Specifically, mIoU quantifies the segmentation accuracy of the model by calculating the average ratio of the intersection to the union of the predicted results and the ground truth labels. mPA measures the recognition accuracy of the model at the pixel level based on the ratio of correctly classified pixels to the total number of pixels. The F1-Score comprehensively integrates Precision and Recall, balancing the model’s detection completeness (minimizing false negatives) and its anti-misjudgment capability (minimizing false positives) for crack targets.

In addition to segmentation accuracy, the lightweight degree and real-time inference capability of the model are also critical assessment dimensions. Model parameters (Parameters, M) are selected to measure the spatial complexity of the model weights. The computation volume (GFLOPs, G) is utilized to measure the time complexity of the model and its consumption of hardware computing power. Frames Per Second (FPS) is employed to measure the actual inference throughput speed of the model on the hardware.

### 5.3. Ablation Study

To deeply investigate the deconstruction and reconstruction capabilities of the proposed Discrete Wavelet Frequency Decoupling (DWFD) module and the Cross-Frequency Guidance Module (CFGM) on heterogeneous chimeric features, an ablation study was designed. By progressively stripping away the core innovative modules and combining them with the Score-CAM (Score-weighted Class Activation Mapping) heat map technique, the working mechanisms of each internal module within the network were quantitatively analyzed.

In the ablation study, we configured the degraded baseline model to retain the same macroscopic encoder–decoder architecture and skip connections as LFDT-Net. However, the DWFD module was removed from the network structure, and conventional 2×2 max pooling was adopted to replace the wavelet transform for downsampling, causing the feature extraction path to degrade into a single-stream structure. Simultaneously, the CFGM was removed. When the feature maps were transmitted between hierarchical levels, they only underwent conventional concatenation and 3×3 convolution, without any cross-band mask denoising or residual edge sharpening.

Based on this foundation, the DWFD and CFGM modules were independently and sequentially added to the baseline model, and their objective metrics were evaluated on the validation set. The quantitative results of the ablation experiments are presented in Table 5.

As shown in Table 5, the ablation study objectively reveals the substantial contributions of each core module to the overall performance of the model. Faced with the highly physically heterogeneous cross-modal fused features, the baseline model (Model A) suffered a distinct baseline collapse due to the high-frequency edge loss and thermal diffusion misleading caused by conventional downsampling, yielding an mIoU of only 76.21%. Upon the independent introduction of DWFD (Model B) or CFGM (Model C), the model achieved gains in physical edge fidelity and background noise suppression, respectively, lifting the mIoU to 78.18% and 79.85%. When the two modules were integrated into the complete LFDT-Net (Model D), the performance exhibited a significant improvement, with the mIoU jumping to 81.65%. Given the extreme foreground–background imbalance of micro-crack targets (typically occupying <2% of the image pixels), this approximately 5% absolute gain over the baseline represents a highly critical breakthrough rather than a trivial fluctuation, effectively reducing the relative segmentation error by over 20%. This proves that the high-fidelity frequency-domain decoupling provided by DWFD and the residual sharpening mechanism of CFGM work in synergy, successfully resolving the cross-modal feature conflicts in dark environments from the underlying architecture, and achieving a dual breakthrough in both crack topological continuity and pixel-level segmentation accuracy.

**Figure 8:**
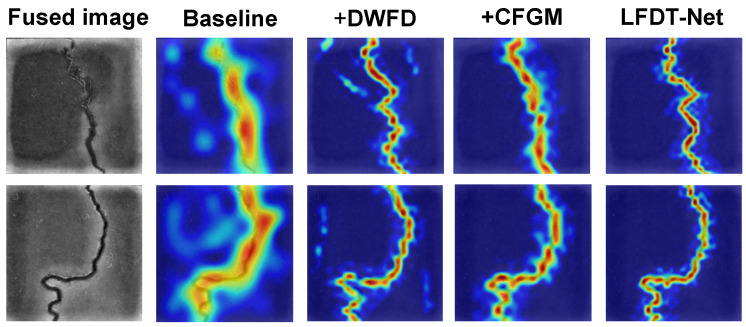
Heatmaps of the ablation study.

To more intuitively reveal the physical effectiveness of the modules, the Score-CAM [29] technique was utilized to extract the class activation heatmaps at the deepest layer of the encoder. In the heatmaps, the red regions represent the high-confidence attention focus during the network’s decision-making process, while the blue regions represent the suppressed background areas. The visual reasoning logic of the four model variants presents extremely significant differences, as shown in Figure 8.

For the baseline model, since conventional pooling operations irreversibly destroy the high-frequency edge information of the visible image, and the network lacks an effective background noise suppression mechanism, its Score-CAM heatmap exhibits a significant feature divergence defect. Upon the introduction of the DWFD module, benefiting from the high-fidelity retention of the visible physical edge features by the wavelet high-pass filters, the high-response regions of the network begin to converge toward the true geometric center of the crack, and the activation band exhibits significant refinement and sharpening characteristics. However, lacking the spatial mask constraints of the CFGM, the high-frequency noise in the background can still penetrate the network layers, resulting in the persistence of some high-confidence, discrete misclassified patches in the non-target regions of the heatmap.

In the architecture where only the CFGM is introduced without high-fidelity frequency-domain decoupling, under the effect of the low-frequency spatial saliency mask, the non-target regions of its Score-CAM heatmap exhibit a uniform low-response state, effectively eliminating the interference of environmental noise. However, because the features have already lost their high-frequency edges due to pooling operations prior to entering this module, the network is unable to generate a high-precision high-frequency sharpening mask. Consequently, although the response of the crack main body is pure, its boundaries still manifest as irregular, blurred transitions.

When the DWFD and CFGM work in synergy to form the complete network, the Score-CAM heatmap exhibits an ideal feature activation distribution. On the one hand, the denoising mechanism of the CFGM thoroughly eradicates the high-frequency background interference in extremely dark environments. On the other hand, relying on the precise high-frequency physical boundaries provided by the DWFD, the residual operator of the CFGM achieves high-precision spatial pruning on the thermal diffusion edges of the infrared features. The ultimately generated heatmap manifests as an energy band with high-response values and complete topological connectivity, achieving a high degree of coincidence with the true crack skeleton. This visualization result intuitively demonstrates that the frequency-domain decoupling and cross-guidance architecture can effectively overcome the physical domain conflicts of dual-modal heterogeneous features in dark environments, achieving high-precision feature reconstruction.

### 5.4. Comparative Experiments

**Figure 9:**
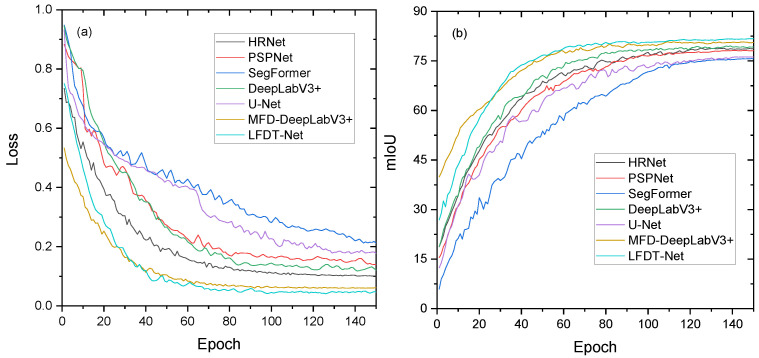
Training dynamics of the compared models: (**a**) training loss convergence curves; (**b**) validation mIoU curves.

To comprehensively evaluate the overall performance of the proposed LFDT-Net in the task of heterogeneous crack segmentation in dark environments, six current mainstream semantic segmentation networks were selected as comparison baselines, namely DeepLabV3+, HRNet, PSPNet, U-Net, SegFormer, and MFD -DeepLabV3+ [30]. All models were trained and tested on the same training and validation sets, and unified hyperparameter configurations were adopted. The experimental results are shown in Table 6. Furthermore, to explicitly demonstrate that all models have been properly trained and to analyze their convergence behaviors, the training loss and validation mIoU curves over 150 epochs are recorded and presented in Figure 9.

As illustrated by the training dynamics in Figure 9, all models exhibit healthy convergence trends without severe overfitting. Notably, the recently proposed MFD-DeepLabV3+ demonstrates a remarkably low initial loss (Figure 9a) and rapid early convergence, benefiting from its transfer learning initialization. Reflected in the final accuracy metrics (Table 6), LFDT-Net achieved optimal results across all segmentation accuracy indicators; its mIoU reached 81.7%, outperforming the recently proposed 2025 SOTA model MFD-DeepLabV3+ (80.6%) by 1.1 percentage points, and the classic DeepLabV3+ (79.2%) by 2.5 percentage points. It also secured the highest mPA (86.5%) and mFscore (85.4%). Considering that micro-cracks occupy an extremely small fraction of the image pixels, this 2.5% absolute performance gain over DeepLabV3+ represents a noticeable and critical improvement in preserving fine topological connectivity rather than a mere statistical fluctuation. This noticeable improvement stems from the high-fidelity frequency decoupling designed for heterogeneous features. General networks (e.g., DeepLabV3+, PSPNet) rely on traditional pooling, which acts as a low-pass filter and irreversibly obliterates visible high-frequency edges. While the recent MFD-DeepLabV3+ mitigates this by using channel attention mechanisms, it still operates within a coupled feature space. In contrast, LFDT-Net mathematically decouples infrared thermal radiation from visible details, preventing thermal diffusion interference at the fundamental architectural level. Other architectures, such as HRNet and SegFormer, despite their high-resolution or global receptive field advantages, lack this physical cross-modal decoupling, limiting their precision on microscopic crack topologies.

In terms of computational efficiency, LFDT-Net strikes an exceptional balance. With a parameter count of 21.5 M and a computational load of 24.5 GFLOPs, it is vastly more lightweight than U-Net (428.6 GFLOPs) and DeepLabV3+ (154.7 GFLOPs). Although MFD-DeepLabV3+ (5.6 M) and SegFormer (3.7 M) possess fewer parameters and higher inference speeds (109.6 and 110.6 FPS, respectively), LFDT-Net still easily satisfies real-time engineering deployment requirements with a rapid 82.6 FPS. This high efficiency is achieved because the Discrete Wavelet Transform replaces computationally heavy convolutional downsampling, compressing feature maps while preserving high-frequency details. Ultimately, LFDT-Net secures the highest segmentation accuracy with a highly competitive operational speed.

**Figure 10:**
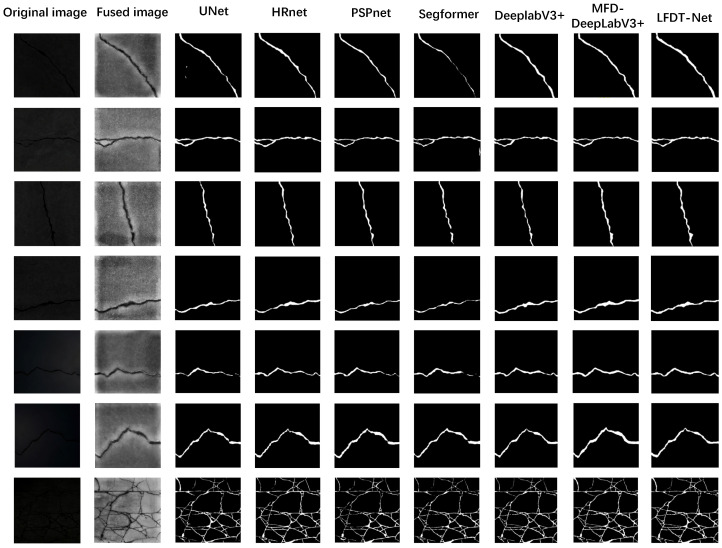
Comparison of segmentation effects of different models.

To intuitively display the pixel-level segmentation performance in dark environments, seven sets of typical complex crack samples from the testing set are visually compared. As shown in Figure 10, from left to right, the figure displays the original extremely dark visible images, the multi-modal fused images processed by the preliminary registration algorithm, and the final output masks of U-Net, HRNet, PSPNet, SegFormer, DeepLabV3+, MFD-DeepLabV3+, and the proposed LFDT-Net.

As can be clearly observed from the comparison of the first two columns in Figure 10, in the original visible images captured in extremely dark environments, the crack targets are almost completely submerged in the background due to a severe lack of illumination. After heterogeneous multi-modal fusion (the second column), the overall orientation and microscopic edge details of the cracks are explicitly revealed. This intuitively proves that the preliminary fusion strategy provides a high-contrast feature base for the deep learning network; however, it still contains inherent infrared thermal distortions and high-frequency dark-light noise.

From the segmentation results, conventional architectures expose distinct vulnerabilities. In the first set of samples, U-Net exhibits discrete false-positive noise, lacking an effective suppression mechanism against dark background noise. Crucially, to explicitly evaluate the performance on real-world fine cracks, the final set of samples introduces a highly challenging scenario that features a dense, intersecting web of micro-cracks. In this specific case, U-Net and the pyramid-pooling-based PSPNet suffer a severe loss of fine details, failing to reconstruct the intersecting web due to the irreversible destruction of high-frequency microscopic edges caused by excessive pooling. SegFormer, despite its global receptive field, exhibits continuity fractures along the slender, thread-like branches. Similarly, while the recent MFD-DeepLabV3+ captures macroscopic branches, it struggles to perfectly preserve the intricate topological intersections under such fine scales.

In contrast, the proposed LFDT-Net demonstrates the most robust segmentation performance across all extreme testing scenarios, including the complex fine-crack web. Under the effect of the DWFD module locking onto visible high-frequency edges and the CFGM removing low-frequency background noise, LFDT-Net contains no stray background noise and successfully reconstructs faint crack tails and fine intersecting networks. However, regarding potential issues in practical deployment, detecting sub-pixel-level extremely fine cracks under profound darkness remains inherently challenging. While LFDT-Net significantly preserves structural continuity, severe thermal diffusion artifacts from the infrared modality can occasionally blur the absolute boundaries of micro-cracks, leading to slight topological disconnections at the most extreme branch tips. Nevertheless, the visual evidence fundamentally confirms the superior accuracy and robustness of the proposed frequency decoupling architecture when handling heterogeneous and fine cracks in dark environments.

## 6. Conclusions

To address the challenges of difficult heterogeneous image registration and low segmentation accuracy, caused by a severe lack of illumination and significant modal differences in concrete cracks in extremely dark environments, this paper proposed a complete two-stage processing framework of fusion and segmentation. The main conclusions are drawn as follows:

(1) In terms of heterogeneous image registration and fusion, a registration algorithm based on morphological priors and multi-level quadtree spatial constraints was proposed. By extracting common crack structures of heterogeneous images through morphological transformations, this algorithm transformed the problem from pixel grayscale matching into spatial topological matching. The multi-level quadtree mechanism was utilized to achieve coarse-to-fine adaptive-control point extraction, effectively overcoming geometric distortions and thermal diffusion effects of infrared images. The fused image synthesizes the high saliency of infrared and high sharpness of visible light, providing a high-quality chimeric feature map for subsequent segmentation.

(2) Regarding semantic crack segmentation, the Latent Frequency-Decoupled Topological Network (LFDT-Net) was proposed. It utilizes the Discrete Wavelet Transform to replace conventional pooling, achieving information-preserving decoupling of the low-frequency backbone (infrared) and the high-frequency edges (visible light). A Cross-Frequency Guidance Module was designed to eliminate the double-edge artifacts caused by microscopic parallax through an interactive mechanism of a low frequency guiding high-frequency denoising, and a high frequency guiding low-frequency sharpening. Furthermore, a skeleton-aware topological loss function was introduced to constrain network learning from the perspective of mechanical continuity, significantly enhancing the topological integrity of crack segmentation.

(3) The experimental results demonstrate that the proposed method performs outstandingly on the self-built heterogeneous multi-modal crack dataset. The ablation study verified the effectiveness of each module, with the complete model achieving a 5.44 percentage point increase in mIoU compared to the baseline. In comparative experiments, LFDT-Net comprehensively outperformed mainstream models such as DeepLabV3+ and U-Net in segmentation accuracy, with mIoU, mPA, and mFscore reaching 81.7%, 86.5%, and 85.4%, respectively. Simultaneously, it possesses favorable computational efficiency (21.5 M parameters, 82.6 FPS), capable of effectively suppressing dark background noise and precisely restoring microscopic edges and continuous topological structures of faint cracks.

(4) Regarding practical implementation and future work, the proposed LFDT-Net demonstrates high computational efficiency (82.6 FPS), readily satisfying the real-time processing demands of mobile inspection robots or UAVs in industrial environments. While the current methodology has been extensively validated on concrete structures, the core physical mechanism of cross-modal frequency decoupling possesses strong theoretical generalizability. Future research will focus on extending this dual-modal framework to other critical engineering materials with distinct thermal-visual signatures, such as asphalt pavements and metallic pipelines, and further compressing the model footprint for direct deployment on resource-constrained edge computing devices.

## Figures and Tables

**Table 1:** Ablation study of fusion weights (α, β) based on IQA metrics.

Weight Combinations	AG	SF	EN
α=1.0,β=1.0	8.42	16.35	5.21
α=0.8,β=1.5	10.15	18.72	5.84
α=0.6,β=1.5	11.24	20.31	6.02
α=0.6,β=2.5	14.56	24.88	6.75
α=0.4,β=3.0	12.80	22.15	6.43

**Table 2:** Quantitative metrics of cross-modal registration and fusion.

Metric Category	Evaluation Metric	Average Value
Registration Accuracy	Root Mean Square Error (RMSE)/pixels	2.24
Fusion Quality	Information Entropy (EN)	6.67
Fusion Quality	Structural Similarity (SSIM)—IR vs. Fused	0.92
Fusion Quality	Structural Similarity (SSIM)—Visible vs. Fused	0.45

**Table 3:** Detailed architecture of the proposed LFDT-Net.

Stage	Component/Layer	Operation Details	Input Dimension	Output Dimension
Input	Initial Fusion Map	-	H×W×1	H×W×1
Encoder Stage 1	Initial Feature Ext	Conv 3×3, BN, ReLU	H×W×1	H×W×64
	DWFD Module	2D-DWT (Haar), Stride 2	H×W×64	H/2×W/2×64 (Dual Stream)
	CFGM	Mask Gen, Element-wise ⊗, Conv 3×3	H/2×W/2×64 (Dual)	$H/2\times W/2\times C_{1}$$\;(C_{1}=64$)
Encoder Stage 2	Feature Ext	Conv 3×3, BN, ReLU	H/2×W/2×64	H/2×W/2×128
	DWFD Module	2D-DWT (Haar), Stride 2	H/2×W/2×128	H/4×W/4×128 (Dual Stream)
	CFGM	Mask Gen, Element-wise ⊗, Conv 3×3	H/4×W/4×128 (Dual)	$H/4\times W/4\times C_{2}$$\;(C_{2}=128$)
Encoder Stage 3	Feature Ext	Conv 3×3, BN, ReLU	H/4×W/4×128	H/4×W/4×256
	DWFD Module	2D-DWT (Haar), Stride 2	H/4×W/4×256	H/8×W/8×256 (Dual Stream)
	CFGM	Mask Gen, Element-wise ⊗, Conv 3×3	H/8×W/8×256 (Dual)	$H/8\times W/8\times C_{3}$$\;(C_{3}=256$)
Encoder Stage 4	Feature Ext	Conv 3×3, BN, ReLU	H/8×W/8×256	H/8×W/8×512
	DWFD Module	2D-DWT (Haar), Stride 2	H/8×W/8×512	H/16×W/16×512 (Dual Stream)
	CFGM	Mask Gen, Element-wise ⊗, Conv 3×3	H/16×W/16×512 (Dual)	$H/16\times W/16\times C_{4}$$\;(C_{4}=512$)
Decoder	Up-Stage 1	Bilinear Upsample ×2, Concat (Stage 3), Conv 3×3	H/16×W/16×512	H/8×W/8×256
	Up-Stage 2	Bilinear Upsample ×2, Concat (Stage 2), Conv 3×3	H/8×W/8×256	H/4×W/4×128
	Up-Stage 3	Bilinear Upsample ×2, Concat (Stage 1), Conv 3×3	H/4×W/4×128	H/2×W/2×64
	Up-Stage 4	Bilinear Upsample ×2, Conv 3×3	H/2×W/2×64	H×W×64
Output	Prediction Head	Conv 1×1, Sigmoid	H×W×64	H×W×1

**Table 4:** Hyperparameter settings.

Hyperparameters	Value
Image Size	500 × 500
Batch Size	16
Epochs	150
Initial LR	1 × 10^−4^
LR Scheduler	Cosine Annealing
Weight Decay	1 × 10^−2^

**Table 5:** Performance comparison of different models.

Model Variants	DWFD	CFGM	mIoU (%)	F1-Score (%)	mPA (%)
Model A (Baseline model)	×	×	76.21	79.84	80.30
Model B (+DWFD)	√	×	78.18	82.92	83.15
Model C (+CFGM)	×	√	79.85	83.53	84.40
Model D (LFDT-Net)	√	√	81.65	85.35	86.52

Note: × indicates that the component is not used; √ indicates that the component is used.

**Table 6:** Comparison test results of different models.

Model	mIoU/%	mPA/%	mFscore/%	Parameters/M	FPS/(f·s^−1^)	Gflops/G
DeepLabV3+	79.2	82.9	83.6	54.7	47.7	154.7
HRnet	78.7	81.5	82.2	9.6	42.8	30.8
PSPNet	78.1	80.8	81.4	46.7	81.1	108.2
Segformer	75.6	78.3	78.9	3.7	110.6	10.6
U-Net	76.2	80.3	79.8	24.9	30.4	428.6
MFD -DeepLabV3+ [30]	80.6	84.7	84.4	5.631	109.6	16.3
LFDT-Net	81.7	86.5	85.4	21.5	82.6	24.5

## Data Availability

The data presented in this study are not publicly available due to confidentiality agreements with the project owner. They are available from the corresponding author upon reasonable request and with permission of the project owner.
